# Assessing racial differences in time to subsequent treatment following androgen deprivation therapy among Veterans with prostate cancer

**DOI:** 10.21203/rs.3.rs-5001707/v1

**Published:** 2024-10-18

**Authors:** Nadine Friedrich, Nadine Friedrich, Jessica Janes, Joshua Parrish, Amanda De Hoedt, Janis Pruett, Mark Fallick, Raj Gandhi, Agnes Hong, Nicholas Tatonetti, Stephen Freedland

**Affiliations:** Cedars-Sinai Medical Center; Cedars-Sinai Medical Center; IMR; Pfizer Inc.; Department of Computational Biomedicine, Cedars-Sinai Medical Center; Cedars-Sinai Cancer Center

**Keywords:** prostate cancer, androgen deprivation therapy, subsequent treatment

## Abstract

**Background:**

For metastatic and certain advanced prostate cancer (PC), guidelines support intensified androgen deprivation therapy (ADT) as first-line (1L) systemic treatment for improved outcomes. However, some patients receive ADT alone, leading to tumor progression requiring 2nd line therapy. Despite significant racial disparities in PC outcomes, there are no population-level studies assessing racial differences in time to subsequent treatment after 1L ADT.

**Methods:**

We performed a retrospective population-level analysis to assess the association between race and time to subsequent treatment after ADT in the Veterans Affairs Health Care System. Primary outcome was time from ADT monotherapy to subsequent treatment, defined as receipt of androgen receptor pathway inhibitor (ARPI), non-steroidal first-generation anti-androgen (NSAA), chemotherapy, or other treatments. We used Cox models and Kaplan-Meier (KM) analyses to estimate subsequent treatment rates by Non-Hispanic White [NHW], Non-Hispanic Black [NHB], Hispanic and Other patients adjusted for baseline covariates.

**Results:**

From 2001–2021, 141,495 PC patients received ADT alone. During median (IQR) follow-up of 51.1 (22.8, 97.2) months, 28,144 patients (20%) had subsequent treatment: 11,319 (40%) ARPIs, 12,990 (46%) NSAAs, 3,402 (12%) chemotherapy and 433 (2%) other 2nd line therapies. NHB had significantly lower subsequent treatment rates (HR = 0.82, 95%CI = 0.80–0.85) compared to NHW. Both Hispanic (HR = 0.93, 95%CI = 0.88–0.98) and Other men (HR = 0.91, 95%CI = 0.84–0.98), also had lower subsequent treatment rates.

**Conclusions:**

All races examined had significantly lower rates of subsequent treatment after 1L ADT relative to NHW. Further investigation is needed to determine if these lower rates of subsequent treatment reflect lower rate of progression or undertreatment of progressing patients.

## INTRODUCTION

Advanced and metastatic prostate cancer (PC) is often treated with androgen deprivation therapy (ADT)^1^, which decreases PSA and slows tumor growth. However, eventually, many patients will progress to castration-resistant PC (CRPC) either as rising PSA or radiographic progression and require a second line of systemic therapy. ^2^. Guideline concordant options for CRPC, depending on tumor metastasis, include chemotherapy and androgen receptor pathway inhibitors (ARPIs; such as abiraterone, apalutamide, darolutamide, and enzalutamide)^3,4^. Other options, like Radium-223 and Sipuleucel-T, are used infrequently as first-line CRPC agents ^5^. Some men with CRPC are also treated with first-generation non-steroidal anti-androgens (NSAA) (i.e. bicalutamide), despite not being guideline concordant^6^. Regardless of the agent used, subsequent treatment following ADT alone typically indicates tumor progression and poor prognosis^7^.

While Black men are more likely to be diagnosed with PC and are nearly two times more likely to die from PC versus white men^8^, whether time to subsequent treatment following ADT initiation differs by race (Non-Hispanic White [NHW], Non-Hispanic Black [NHB], Hispanic and Other) is unknown. To examine this, we used the Veterans Affairs (VA) health care system (VAHCS) data to perform a retrospective population-level analysis assessing the association between race and subsequent treatment after ADT. Based on previous studies from our team, which showed no difference in time to metastasis after ADT^9^, we hypothesized time to subsequent treatment would be similar across races.

## MATERIALS AND METHODS

### Design & Cohort

After IRB approval, we performed a nationwide retrospective study using the VA Informatics and Computing Infrastructure (VINCI), an analytic platform with access to all electronic health record (EHR) data in the Veterans Affairs Health Care System. We utilized specific queries within VINCI to identify male veterans who were diagnosed with PC between 2001–2021 and were ≥ 18 years old at diagnosis. We limited queries to individuals who were considered active VA users (defined as ≥ 2 visits within the 5 years of the study period). Demographic and clinical data were extracted from VINCI, including first receipt of each of the following PC-specific treatments: bilateral orchiectomy, radiation, radical prostatectomy, ADT, ARPIs (abiraterone, apalutamide, darolutamide, and enzalutamide), NSAAs (bicalutamide, flutamide, and nilutamide), chemotherapy (cabazitaxel, docetaxel), and other systemic therapies (olaparib, pembrolizumab, radium-223, sipuleucel-T).

**Figure 1:**
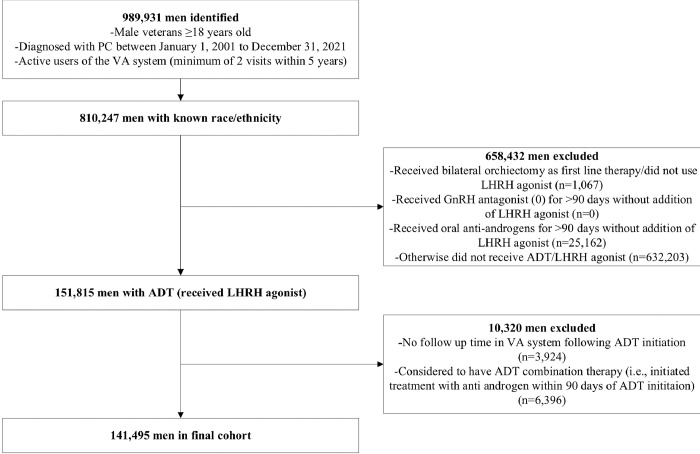
Consort Diagram Abbreviations: PC= Prostate Cancer; VA= Veterans Affairs; ADT= Androgen Deprivation Therapy; LHRH= Luteinizing-hormone releasing hormone; GnRH= gonadotropin-releasing hormone.

Only patients who received ADT, defined as luteinizing-hormone releasing hormone (LHRH) agonist (Leuprolide, Goserelin, Triptorelin), as 1L systemic therapy for PC were included. The number of men who received LHRH antagonists was small and therefore these patients were excluded. Patients may have received prior local therapy for PC. Patients may also have received an NSAA for up to 90 days for blocking of testosterone flare, but patients who stayed on NSAA for ≥ 90 days were considered on combined androgen blockade and were excluded. We also excluded patients who received a second systemic treatment initiated within 90 days after ADT start. Patients were excluded if they were missing pertinent data on race or ethnicity or had no follow-up after ADT start (Fig. 1).

### Study Variables

Race and ethnicity were both self-reported and combined into a single variable with categories of NHW, NHB, Hispanic (regardless of race), or Other. Other included Asian, Biracial, Native Hawaiian/Other Pacific Islander, and Native American/Alaskan Native races of Non-Hispanic ethnicity. Age at ADT and year of ADT were captured and treated as continuous variables. Body Mass Index (BMI) was derived using the weight closest to, but prior to ADT initiation, and median height across all heights observed. BMI was categorized as < 25, 25–29, ≥ 30, or unknown if missing either height or weight. Comorbidities defined under the Charlson Comorbidity Index (CCI) were identified from claims data (ICD 9/10 codes) and summed to the day before the ADT start (index date). CCI was categorized as 0, 1, 2, or 3+. Clinical stage and Gleason grade were obtained from the cancer registry, these data were absent for most patients and, therefore, not used in the analysis. All PSA and testosterone measurements were captured, excluding values above or below 3 standard deviations. Baseline PSA and testosterone variables were identified as the value closest to but prior to ADT start. Both PSA and testosterone were captured as continuous variables but analyzed as categorical variables to include those with unknown values. PSA was categorized as < 4, 4–10, 10.1–19.9, ≥ 20, or unknown, and testosterone was categorized into quartiles or unknown. Receipt of radiation or radical prostatectomy prior to ADT was both categorized as yes versus no. Number of months from PC diagnosis to ADT initiation was computed.

Our primary outcome was time from ADT to subsequent treatment, defined as (1) receipt of add-on ARPI therapy (Abiraterone, Apalutamide, Darolutamide, Enzalutamide) or other systemic 2nd line therapy (Olaparib, Pembrolizumab, Radium-223, Sipuleucel-T), (2) addition of a NSAA therapy, or (3) receipt of chemotherapy (cabazitaxel, docetaxel). If patients received multiple treatments, earliest date was used. Patients with no evidence of subsequent treatment were censored at time of death or last known visit within the VA system.

### Statistical Analysis

Baseline demographic and clinical characteristics were summarized at time of ADT using median, interquartile range, and range for continuous variables and frequencies and percentages for categorical variables. Differences in characteristics between race/ethnicity groups were assessed using Kruskal-Wallis tests for continuous variables and Chi square tests or Fisher’s Exact tests where appropriate for categorical variables.

Kaplan-Meier (KM) curves for time from ADT to subsequent treatment were stratified by race/ethnicity and point estimates for the proportion free from subsequent treatment at 3, 5, 7, 10, and 15 years post-ADT were obtained. A log-rank test was used to test for differences in curves between groups. Univariable and multivariable Cox proportional hazards models assessed the association between race/ethnicity and time to subsequent treatment. Candidate variables for inclusion in the multivariable models included race/ethnicity, age at ADT, year of ADT, time from PC diagnosis to ADT, CCI, BMI, PSA and testosterone levels prior to ADT, radiation therapy, and radical prostatectomy prior to ADT. Variables that were selected a priori in univariable analysis were included in the multivariable model. We followed the rule of thumb to only include 1 predictor for every 10 events observed to reduce the likelihood of overfitting. If the model needed to be reduced, comparisons between variations of the model were assessed with model fit indices such as Bayesian information criterion (BIC) and Akaike information criterion (AIC) to determine the best fitting model.

Collinearity between variables in the multivariable model was checked and variance inflation factors were assessed. Assumptions of linearity and proportional hazards were assessed with standard methods such as plotting Martingale and Schoenfeld residuals by time. The final model included all candidate variables except prior receipt of radical prostatectomy which was collinear with PSA and added no additional predictive value. Interactions between race and covariates were tested in multivariable analysis. Due to significant interactions, we also stratified the analysis by race/ethnicity to assess associations between covariates and subsequent treatment separately in each group. For exploratory purposes, number of treatment events in each group were categorized by type (ARPI therapy, other systemic 2nd line therapy, NSAA, or chemotherapy)

All statistical analyses were performed with SAS Enterprise Guide 8.2. Statistical significance was predetermined at p < 0.05.

## RESULTS

A total of 989,931 PC patients were identified, of which 810,247 had known race and ethnicity. Among these, 151,815 received ADT as a 1L systemic therapy at some point during their PC journey. After excluding those who initiated ADT on the same date as their last known visit to the VA along with those who received ADT combined with another anti-PC agent defined as receiving another anti-PC agent within 30 days before or 90 days after ADT start, the study cohort consisted of 141,495 patients.

Of this cohort, 94,500 (67%) were NHW, 36,421 (26%) NHB, 7,287 (5%) Hispanic, and 3,287 (2%) Other.

### Patient Baseline Demographics and Clinical Characteristics by Race/Ethnicity

NHB patients were the youngest at ADT initiation [Median (Q1-Q3) = 68.1 (62.3–74.8)] (p < 0.001), received ADT in later years of the study [Median (Q1-Q3) = 2012 (2007–2017)] (p < 0.001), had the highest PSA levels prior to ADT initiation [Median (Q1-Q3) = 10.4 (5.2–25.2)] (p < 0.001), the highest number with 3 or more comorbidities prior to ADT initiation (46%)(p < 0.001), and the highest rate of radiation use prior to ADT (10%)(p < 0.001) compared to all other groups (Table 1). Hispanic patients had the highest testosterone levels prior to ADT initiation [Median (Q1-Q3) = 315 (210–424)] (p < 0.001), the highest proportion of those with BMI range 25–29 (42%) (p < 0.001), and the longest follow-up [Median (Q1-Q3) = 58.4 (25.2, 109.0)] (p < 0.001) compared to all other groups.

### Time to subsequent treatment and KM estimates by race/ethnicity

**Figure 2:**
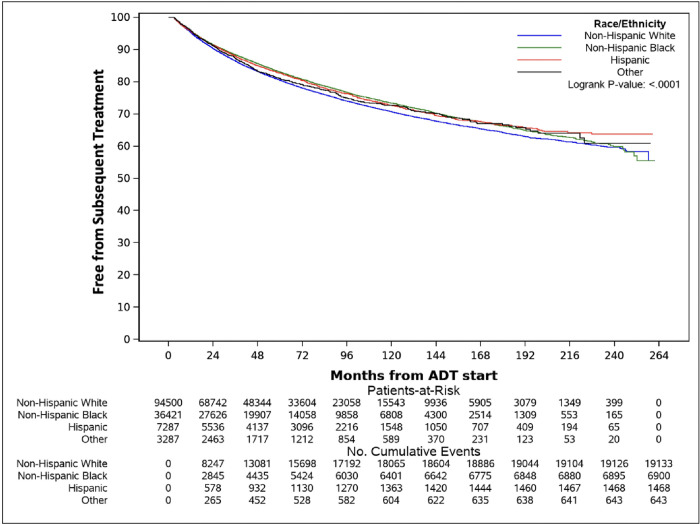
Kaplan-Meier curve for time to subsequent treatment stratified by race Abbreviations: ADT= Androgen Deprivation Therapy.

With a median (Q1-Q3) follow-up of 51.1 (22.8–97.2) months, 28,144 (20%) subsequent treatment events were observed across all races. Among NHWs, 19,133/94,500 (20.2%) events were observed compared to 6,900/3,6421 (18.9%) for NHBs, 1,468/7,287 (20.1%) for Hispanics, and 643/3,287 (19.6%) for Others (Table 2). NHWs were most likely to have subsequent treatment over time (log rank p-value < 0.001) (Fig. 2). The 3- and 5- year estimates (95% CI) of being subsequent treatment-free were 86.5% (86.2%–86.7%) and 80.5% (80.2%–80.8%) respectively for NHWs compared to 88.4% (88.0%–88.7%) and 83.0 (82.6–83.5%) respectively for NHBs.

### Univariable and multivariable associations with time to subsequent treatment

In univariable analysis, NHBs experienced significantly lower rates of subsequent treatment [HR (95% CI): 0.89 (0.86, 0.91)] compared to NHWs, as were Hispanics [HR (95% CI): 0.90 (0.86, 0.95)] (Table 3). Others were also at lower rate than NHWs but not significantly so [HR (95% CI): 0.94 (0.87, 1.01)]. In multivariable analysis, all races were at significantly lower rates for escalating treatment compared to NHWs, including NHBs [HR (95% CI): 0.82 (0.80, 0.85)], Hispanics [HR (95% CI): 0.93 (0.88, 0.98)], and Others [HR (95% CI): 0.91 (0.84, 0.98)]. However, subsequent treatment was lowest in NHBs (lowest HR relative to NHWs).

### Subsequent treatment events broken down by type of treatment category

Among all 28,144 subsequent treatment events, 12,990 (46%) were NSAA, 11,319 (40%) were ARPIs, 3,402 (12%) were chemotherapy, and 433 (2%) were other systemic 2nd line therapies (Table 4) While the rate of ARPIs between NHBs and NHWs was similar (41% vs. 40%, respectively), Hispanics had the lowest proportion (35%). Hispanics had the highest (51%) proportion of events that were NSAA followed by NHWs (47%), while NHBs were most likely to get chemotherapy (15%) as subsequent treatment, followed by Hispanics (13%) and NHWs (11%).

## DISCUSSION

Many patients with advanced and metastatic PC will progress to subsequent treatment after ADT initiation^9^. Whether race and ethnicity are associated with the time to subsequent treatment after 1L ADT was previously unknown. To address this, we performed a population-level analysis using retrospective data from the nationwide VAHCS to compare racial differences (NHBs, NHWs, Hispanics, and Others) in time to subsequent treatment after 1L ADT. Using a cohort of over 140,000 patients, we found all races were less likely to receive subsequent treatment relative to NHWs, with NHBs having the lowest subsequent treatment rates. Whether this represents better cancer control or undertreatment of patients who do progress remains to be determined.

In patients with metastatic PC and even in certain circumstances for advanced PC (localized very high risk with radiation; biochemical recurrence with short PSA doubling time), guidelines and clinical trial data universally support intensified ADT as this improves long-term outcomes. However, despite these guidelines, a subset of patients will receive ADT alone^10^. Moreover, historically (i.e. the period covered during this study), intensified ADT was not routinely recommended as the seminal studies had not yet been conducted showing the benefits of intensified ADT. Typically, PC progresses to CRPC within an average of 2–3 years, necessitating subsequent treatment to effectively manage the disease^7^. As such, subsequent treatment can be viewed as a sign that the initial treatment is no longer effective. Subsequent treatment options include but are not limited to chemotherapy, ARPIs, or NSAAs, all with the goal of slowing cancer progression, extending survival, and improving quality of life^11^. In real-world studies, where capturing disease progression is challenging, time to next treatment is often used as an intermediate endpoint^12,13^. However, delayed subsequent treatment can reflect either better tumor control (i.e., no need for subsequent treatment) or undertreatment of patients who do progress. In large population-based claims studies, distinguishing between these possibilities is difficult. Nonetheless, subsequent treatment remains an important clinical endpoint as it signals a step-up in care with potential associated side-effects. To date, few studies examined race as a prognostic factor for subsequent treatment.

The effectiveness of ADT in managing PC may vary across races. Vidal et al examined the relationship between race and metastases development in men receiving ADT after non-metastatic biochemical recurrence following radical prostatectomy^9^. Outcomes were comparable between White and Black individuals, suggesting race didn’t significantly influence the risk of metastases in this population. However, the study included a modest sample size, thus findings should be interpreted with caution. Similarly, a recent systematic review of men with metastatic Castration-Sensitive Prostate Cancer (mCSPC) treated with ADT alone (with or without NSAA), found similar survival outcomes between Whites and Blacks^14^. Nonetheless, some studies found poorer survival in mCSPC for Black men vs. White^15,16^. Notably, among the patient population newly starting ADT, no studies that we are aware of, show *better* outcomes among Black men. As such, it is intriguing that while we found that rates of subsequent treatment were lower for NHB men, the exact causes of this are unknown. Possibly, this may reflect undertreatment of Black men when they progress, as prior studies in both Medicare and the VA populations have demonstrated ^10,17,18^. Alternatively, our results may reflect better tumor control in Black men relative to White men. While intriguing, there are no current data suggesting improved outcomes among Black men with PC treated with ADT alone vs. White men. However, our large sample size, allowed us to detect modest differences in subsequent treatment. Thus, prior studies assessing outcomes by race may have been underpowered to detect improved tumor control among Black men. Therefore, we cannot conclude with certainty whether our results reflect undertreatment and/or improved outcomes among Black men and this requires further study using other surrogate endpoints for tumor control.

Subsequent treatment rates were also lower among Hispanics and individuals from other racial or ethnic backgrounds compared to NHWs. We are not aware of data specifically examining ADT and tumor control across Hispanic and Other races. However, the same recent systematic review of men with metastatic PC found no significant difference in overall survival between Hispanics and NHW men with PC^14^. Similar to NHB men outlined above, the absence of data suggesting differences in tumor control with ADT suggests that the lower rates of subsequent treatment may reflect undertreatment of recurrent disease. Notably, prior studies, due to limited sample sizes, may not have been powered to detect the modest associations seen in our study. Intriguingly, literature suggests Black men have better outcomes with other systemic treatments for PC such as chemotherapy or novel hormonal therapies^19–21^. As such, future studies are needed to assess ADT efficacy across races.

One of the notable strengths of this study is its substantial sample size of all races, which enhances the statistical power and robustness of the findings. The inclusion of a large number of NHBs individuals, often underrepresented in PC studies, improves the generalizability of the results. Moreover, a notable strength of our study is the VA’s equal access setting and low-cost medications reduce socioeconomic barriers to care.

Despite these strengths, the study has limitations. First, we were unable to isolate the precise reasons for subsequent treatments. Second, we did not capture any other measures of oncological control (PSA response, time to metastasis) which could have provided evidence to support or contradict the hypothesis that the lower rates of subsequent treatments may be linked to better cancer control. Patients were included from 2001, before current therapies (e.g., abiraterone, enzalutamide) were available, which likely explains the high rate of NSAA use as the next treatment. Finally, we lacked information on PC prognostic factors (stage, grade, disease status) to include in our multivariable models.

## CONCLUSIONS

This is the first population-level study assessing racial differences in time to subsequent treatment of men receiving 1L ADT. We found that all races had a significantly lower rate of subsequent treatment relative to NHWs.

Reasons for this variation in practice are unknown, highlighting the need for additional research on how patients should receive timely and appropriate care throughout their PC treatment.

## Figures and Tables

**Table 1:** Characteristics of patients who received ADT stratified by race/ethnicity (N = 141,495)

	Non-Hispanic White (N = 94,500)	Non-Hispanic Black (N = 36,421)	Hispanic (N = 7,287)	Other (N = 3,287)	p value
**Age at ADT start**					< 0.001
Median	73.0	68.1	73.4	71.5	
Q1, Q3	66.9, 79.8	62.3, 74.8	67.1, 79.2	65.4, 78.0	
Range	(36.2–104.2)	(38.1–104.9)	(39.7–99.8)	(39.2–101.7)	
**Year of ADT start**					< 0.001
Median	2011	2012	2011	2012	
Q1, Q3	2006, 2016	2007, 2017	2006, 2016	2007, 2017	
Range	(2001–2022)	(2001–2022)	(2001–2022)	(2001–2021)	
**PSA (ng/ml) at ADT start**					< 0.001
Missing	13928	3228	719	484	
Median	7.9	10.4	8.2	8.5	
Q1, Q3	3.3, 18.2	5.2, 25.2	4.0, 18.3	4.0, 19.2	
Range	(0.0–614.0)	(0.0–614.0)	(0.0–606.7)	(0.0–610.0)	
**PSA (ng/ml) at ADT start**					< 0.001
<4.0	22137 (23%)	6158 (17%)	1642 (23%)	697 (21%)	
4.0–10.0	24860 (26%)	9966 (27%)	2153 (30%)	863 (26%)	
10.1–19.9	15085 (16%)	6979 (19%)	1260 (17%)	568 (17%)	
≥20.0	18490 (20%)	10090 (28%)	1513 (21%)	675 (21%)	
Unknown	13928 (15%)	3228 (9%)	719 (10%)	484 (15%)	
**Testosterone level at ADT start**					< 0.001
Missing	84401	31036	6444	2883	
Median	281.3	301.0	315.0	288.1	
Q1, Q3	174.0, 410.0	200.0, 428.0	210.0, 424.0	163.5, 408.9	
Range	(0.0–5800.0)	(0.0–1500.0)	(0.0–1419.0)	(1.6–1500.0)	
**CCI at ADT start**					< 0.001
0	26148 (28%)	8125 (22%)	1627 (22%)	797 (24%)	
1	18638 (20%)	6870 (19%)	1464 (20%)	640 (19%)	
2	13114 (14%)	4773 (13%)	976 (13%)	467 (14%)	
3+	36600 (39%)	16653 (46%)	3220 (44%)	1383 (42%)	
**BMI at ADT start**					< 0.001
Missing	16362	5532	1196	611	
<25	18999 (24%)	9292 (30%)	1745 (29%)	736 (28%)	
25–29	30854 (39%)	10770 (35%)	2541 (42%)	1021 (38%)	
≥30	28285 (36%)	10827 (35%)	1805 (30%)	919 (34%)	
**Months from PC diagnosis to ADT start**					< 0.001
Median	2.8	3.0	3.0	3.0	
Q1, Q3	0.8, 21.4	1.1, 15.3	1.0, 14.9	0.9, 17.9	
Range	(0.0–250.0)	(0.0–243.8)	(0.0–234.4)	(0.0–246.1)	
**RP prior to ADT start?**					< 0.001
No	89927 (95%)	34092 (94%)	6835 (94%)	3118 (95%)	
Yes	4573 (5%)	2329 (6%)	452 (6%)	169 (5%)	
**Radiation prior to ADT start?**					< 0.001
No	88222 (93%)	32837 (90%)	6677 (92%)	3026 (92%)	
Yes	6278 (7%)	3584 (10%)	610 (8%)	261 (8%)	
Follow up*					< 0.001
Median	49.4	53.8	58.4	50.9	
Q1, Q3	21.9, 94.5	24.8, 101.4	25.2, 109.0	23.9, 99.1	
Range	(0.0–262.0)	(0.0–261.7)	(0.0–260.8)	(0.0–259.4)	

* Number of months from ADT to subsequent treatment or to censor date if no subsequent treatment. Across all patients the median (Q1, Q3) was 51.1 (22.8–97.2) months. Overall follow up to death or censor date was 59.6 (29.3–107.0) months. Abbreviations: ADT = Androgen Deprivation Therapy; PSA = Prostate Specific Antigen; CCI = Charlson Comorbidity Index; BMI = Body Mass Index; PC = Prostate Cancer; RP = Radical Prostatectomy.

**Table 2:** Subsequent treatment events (28,144/141,495) and Kaplan-Meier point estimates for the percent free from subsequent treatment by race

		Point Estimates (95% CI)				
Race/Ethnicity	Event/Total	3 years	5 years	7 years	10 years	15 years
Non-Hispanic White	19133/94500	86.5 (86.2–86.7%)	80.5 (80.2–80.8%)	76.0 (75.6–76.3%)	70.7 (70.3–71.1%)	64.2 (63.7–64.8%)
Non-Hispanic Black	6900/36421	88.4 (88.0–88.7%)	83.0 (82.6–83.5%)	78.7 (78.2–79.2%)	73.4 (72.8–74.0%)	66.3 (65.4–67.2%)
Hispanic	1468/7287	87.9 (87.1–88.7%)	82.6 (81.7–83.6%)	78.0 (76.9–79.2%)	72.6 (71.3–74.0%)	66.4 (64.7–68.2%)
Other	643/3287	87.3 (86.1–88.6%)	81.0 (79.5–82.6%)	77.4 (75.6–79.1%)	72.7 (70.7–74.8%)	66.6 (63.8–69.5%)

Note: the KM median was not reached for any group during the study period.

**Table 3:** Univariable and multivariable associations with time to subsequent treatment (N = 141,495)

	Univariable			Multivariable*		
Variable	HR	95% CI	p-value	HR	95% CI	p-value
Race/Ethnicity			<0.001			<0.001
Non-Hispanic White		Ref.			Ref.	
Non-Hispanic Black	0.89	(0.86, 0.91)		0.82	(0.80, 0.85)	
Hispanic	0.90	(0.86, 0.95)		0.93	(0.88, 0.98)	
Other	0.94	(0.87, 1.01)		0.91	(0.84, 0.98)	

* Multivariable model adjusted for age, year of ADT start, months from PC diagnosis to ADT start, CCI, PSA, testosterone level, BMI, and prior radiation treatment. Abbreviations: ADT = Androgen Deprivation Therapy; PSA = Prostate Specific Antigen; CCI = Charlson Comorbidity Index; BMI = Body Mass Index; PC = Prostate Cancer.

**Table 4.** Subsequent treatment events (N = 28,144) broken down by type of treatment category

	Overall (N = 28,144)		Non-Hispanic White (19,133)		Non-Hispanic Black (6,900)		Hispanic (1,468)		Other(N = 643)	
Subsequent treatment Type	n	%	n	%	n	%	n	%	n	%
**Chemotherapy**	3402	12.1	2116	11.1	1041	15.1	183	12.5	62	9.6
**ARPI**	11319	40.2	7682	40.2	2851	41.3	512	34.9	274	42.6
**NSAA**	12990	46.2	9055	47.3	2886	41.8	752	51.2	297	46.2
**Other**	433	1.5	280	1.5	122	1.8	21	1.4	10	1.6

Abbreviations: ARPI = Androgen Receptor Pathway Inhibitors; NSAA = Non-Steroidal Anti-Androgens

## Abbreviations

PC: Prostate Cancer
VA: Veterans Affairs
ADT: Androgen Deprivation Therapy
LHRH: Luteinizing-hormone releasing hormone
GnRH: gonadotropin-releasing hormone
